# Impact of tamoxifen on adipocyte lineage tracing: Inducer of adipogenesis and prolonged nuclear translocation of Cre recombinase

**DOI:** 10.1016/j.molmet.2015.08.004

**Published:** 2015-08-29

**Authors:** Risheng Ye, Qiong A. Wang, Caroline Tao, Lavanya Vishvanath, Mengle Shao, Jeffery G. McDonald, Rana K. Gupta, Philipp E. Scherer

**Affiliations:** 1 Touchstone Diabetes Center, Department of Internal Medicine, The University of Texas Southwestern Medical Center, Dallas, TX, USA; 2 Department of Molecular Genetics, The University of Texas Southwestern Medical Center, Dallas, TX, USA; 3 Department of Cell Biology, The University of Texas Southwestern Medical Center, Dallas, TX, USA

**Keywords:** Tamoxifen, Cre recombinase, Adipose tissue, Lineage tracing

## Abstract

**Background:**

The selective estrogen receptor modulator tamoxifen, in combination with the Cre-ER^T2^ fusion protein, has been one of the mainstream methods to induce genetic recombination and has found widespread application in lineage tracing studies.

**Methods & results:**

Here, we report that tamoxifen exposure at widely used concentrations remains detectable by mass-spectrometric analysis in adipose tissue after a washout period of 10 days. Surprisingly, its ability to maintain nuclear translocation of the Cre-ER^T2^ protein is preserved beyond 2 months of washout. Tamoxifen treatment acutely leads to transient lipoatrophy, followed by *de novo* adipogenesis that reconstitutes the original fat mass. In addition, we find a “synthetically lethal” phenotype for adipocytes when tamoxifen treatment is combined with adipocyte-specific loss-of-function mutants, such as an adipocyte-specific PPARγ knockout. This is observed to a lesser extent when alternative inducible approaches are employed.

**Conclusions:**

These findings highlight the potential for tamoxifen-induced adipogenesis, and the associated drawbacks of the use of tamoxifen in lineage tracing studies, explaining the discrepancy in lineage tracing results from different systems with temporal control of gene targeting.

## Highlights

• Tamoxifen treatment induces acute fat loss and *de novo* adipogenesis in mice.
• PPARγ knockout exacerbates tamoxifen-induced lipoatrophy.
• Tamoxifen-driven Cre-ER^T2^ nuclear translocation lasts beyond two months in adipose.
• *De novo* adipogenesis upon cold exposure is confirmed with the mTmG reporter.

## 1 Introduction

Recent lineage tracing studies have broadly employed inducible recombinase systems, in which the pulse-chase strategies and interpretation of results critically rely on the accurate temporary control of recombinase activity. Examples include Cre recombinase activity that can be modulated by tetracycline-regulated gene expression or tamoxifen-driven nuclear translocation [1].

The estrogen receptor (ER) ligand tamoxifen has been used as an effective drug for ER-positive breast cancers for decades. As selective ER modulators, tamoxifen and its active metabolite, 4-hydroxytamoxifen, function as agonists or antagonists differentially in various tissues [2]. Taking advantage of its ability to induce nuclear translocation of Cre recombinase fused to the relevant ER domains, tamoxifen confers temporal control for genomic manipulations [3], [4]. Combined with supraphysiological doses of tamoxifen, this system has been widely adopted for lineage tracing studies [1]. However, the time frame of tamoxifen activity after washout has not been rigorously defined in adipocytes. Concerns about its ability to exert precisely titrated temporal control over nuclear Cre activity in other tissues have been previously raised by elegant work from the Powers group [5].

Meanwhile, estrogen signaling plays an important role in body fat distribution and metabolism [6]. Clinically, estrogen mitigates obesity, and favors fat deposition in subcutaneous adipose as well as lipolysis in visceral adipose [7]. These actions are effected mainly through direct activation of ERα [8]. A number of tissue-specific knockouts have been described for ERα. In the context of our results here, the inducible ERα knockout in adipocytes is particularly relevant [9]. Mature adipocytes lacking ERα demonstrate increased markers of fibrosis and inflammation, surprisingly also in males. ERα action in adipocytes therefore exerts important functions and protects against adiposity, inflammation and fibrosis in both males and females.

Here we report that tamoxifen administration results in acute loss of fat mass, which can be further exacerbated by PPARγ ablation. After tamoxifen withdrawal, fat pads are restored at least partially due to a wave of *de novo* adipogenesis. This turnover of adipocytes is more prominently seen in the epididymal white adipose tissue (eWAT) compared to the subcutaneous one (sWAT). Importantly, both tamoxifen and 4-hydroxytamoxifen remain both detectable by liquid chromatography–mass spectrometry in adipose tissue 10 days after cessation of treatment, a time conventionally used for pulse-chase type experiments in the context of lineage tracing approaches. The Cre-ER^T2^ protein is visible by immunofluorescence in the nucleus after 2 months. Taken together, our findings shed light on the profound effects of tamoxifen exposure on adipose physiology and draw attention to the use of tamoxifen in adipocyte lineage tracing studies, which has previously given rise to findings inconsistent with other systems. As a recent example, the tetracycline-inducible system suggested *de novo* recruitment of precursor cells during cold-induced “beiging” of inguinal adipose tissue [10]. In contrast, a tamoxifen-based system suggested a model more consistent with trans-differentiation of pre-existing cells [11].

## 2 Materials and methods

### 2.1 Mice

Mice were maintained in 12-hr dark/light cycles, with *ad libitum* access to diet and water. The mouse strains *TRE-Cre* [12], *PPARγ*^*flox/flox*^ [13], *Adn-Cre-ER*^*T2*^ [14], and *Rosa26-mT/mG* [15] were obtained from the Jackson Laboratory. *Adn-rtTA* [16] and AdipoChaser mice [10] were generated and characterized by our laboratory. All mice were bred in the C57BL/6 genetic background. Adult male mice were used unless indicated. Mice were fed on regular (LabDiet #5058) or doxycycline chow diet (600 mg/kg, Bio-Serv #S4107). For cold exposure, mice were housed individually in a 6 °C cold cabinet as previously described [10]. All protocols for mouse use and euthanasia were reviewed and approved by the Institutional Animal Care and Use Committee of the University of Texas Southwestern Medical Center.

### 2.2 Tamoxifen treatment

Tamoxifen solution (20 mg/mL in sunflower oil) was administrated to mice at 100 mg/kg BDW/day for 5 consecutive days, via oral gavage or intraperitoneal injection. For vehicle control mice, sunflower oil was administrated at a matched volume (5 mL/kg BDW/day). At the end point of experiment, mice were euthanized and dissected for both sides of inguinal subcutaneous and epididymal white fat pads. Fat pads were weighted and then processed for paraffin sections as described [17].

### 2.3 Histology

Paraffin sections were subjected to H&E staining in University of Texas Southwestern Medical Center Molecular Pathology Core. Primary antibodies used for immunofluorescence include: perilipin (Fitzgerald #20R-PP004, or Dr. Andy Greenberg, Tufts University), Cre (Millipore #69050-3), and GFP (Abcam #ab13970). Bright field and fluorescence images were acquired on an Olympus FSX100 all-in-one microscope or a Zeiss Axio Observer Z1 inverted microscope.

### 2.4 RT-qPCR

Total RNA from adipose tissue was extracted, subjected to cDNA synthesis and real-time quantitative PCR as previously described [18].

### 2.5 LacZ staining of adipose tissue

The fat pads from AdipoChaser mice were stained for β-galactosidase activity as previously described [10].

### 2.6 Mass spectrometry

Tamoxifen and 4-hydroxytamoxifen were measured by laser capture microdissection and mass spectrometry as previous described [19].

### 2.7 Statistical analysis

Two-tailed student's t-test was applied for all pairwise comparisons. Statistical significance was accepted at p < 0.05.

## 3 Results

### 3.1 Tamoxifen treatment induces acute death of adipocytes

**Figure 1:**
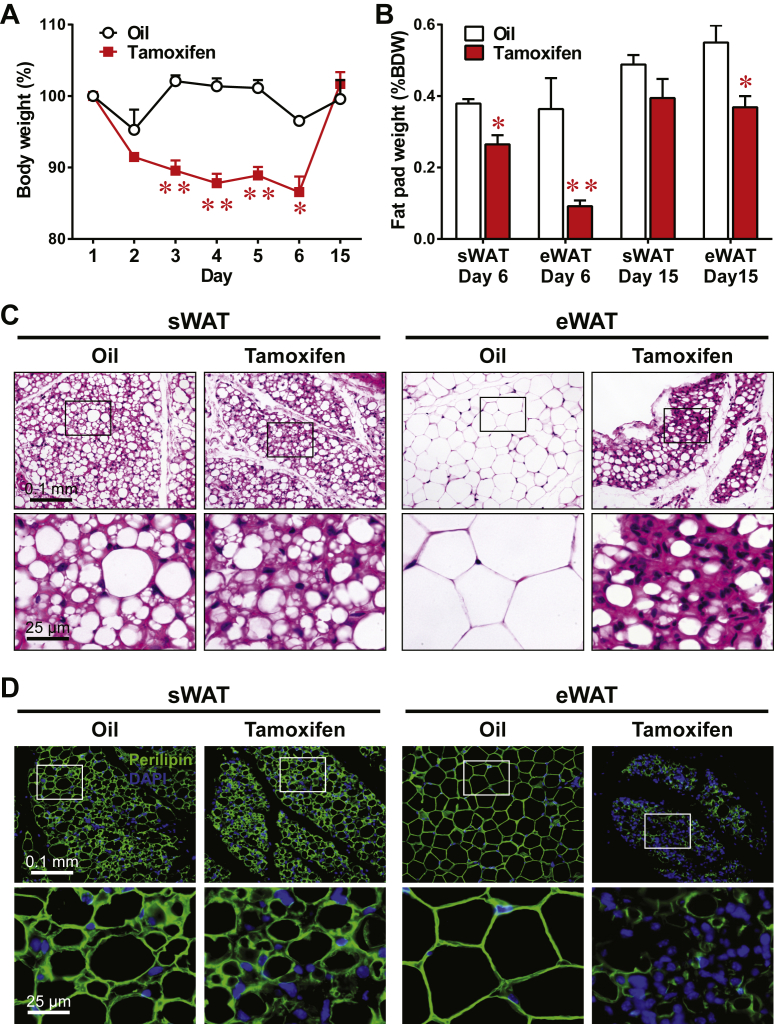
**Tamoxifen treatment induces acute lipoatrophy in mice**. Mice were subjected to 5 consecutive daily oral gavages of tamoxifen (100 mg/kg BDW/day) or vehicle oil at a matched volume (5 mL/kg BDW/day). **(A)** On days 1, 2, 3, 4, 5, 6, and 15, body weight normalized as the percentage of the initial one before the first tamoxifen administration (day 1). **(B)** Fat pad weight normalized against body weight on day 6 or 15. Both sides of inguinal subcutaneous (sWAT) and epididymal white adipose tissue (eWAT) were measured and averaged. Data are presented as the mean ± SEM. n = 5 (oil) and 9 (tamoxifen) on days 1–5. n = 2 and 4 on day 6. n = 3 and 5 on day 15. *p < 0.05, **p < 0.01. **(C and D)** Representative H&E stains **(C)** and perilipin immunofluorescence **(D)** of sWAT and eWAT on day 6. Lower panels show magnified views of the boxed areas in the corresponding upper panels. Non-specific fluorescence level in the negative control was uniformly subtracted from all the images.

To investigate whether tamoxifen treatment imposes significant changes on adipose tissue, we subjected C57BL/6 mice to 5 consecutive daily oral gavages of tamoxifen, at the dose of 100 mg/kg body weight (BDW) per gavage. This treatment protocol is typically used in lineage tracing studies involving adipose tissues [11]. We monitored these mice carefully while treating them as well as during the period immediately after the treatment. These mice exhibited a continuous weight loss following tamoxifen administration (Figure 1A). One day following the last oral gavage (day 6), their body weights had dropped by 13% compared to the initiation of the gavage protocol. In contrast, the vehicle oil-treated mice showed no significant change throughout the treatment period. Ten days after the last tamoxifen treatment (day 15), the mice had restored their body weights to a similar level as the vehicle controls, reflecting only a transient tamoxifen-induced effect on weight loss. This is also a likely explanation why this effect often goes unnoticed.

Immediately after the last day of tamoxifen treatment (day 6), there was a significant reduction in the weight of both the inguinal sWAT and eWAT compared to vehicle controls (Figure 1B), consistent with the overall weight loss. Interestingly, eWAT displayed a much more dramatic decrease than sWAT (75% decrease versus 30% decrease). On day 15, after 10 days of recovery, the sWAT weights in the tamoxifen-treated mice were no longer significantly different from the vehicle controls (p = 0.25). However, eWAT sustained a 33% lower weight (p = 0.02). These results imply that tamoxifen exerts differential effects on different fat pads.

We next examined how tamoxifen induces weight loss in fat pads. Consistent with the weight data, on day 6, sWAT from tamoxifen-treated mice showed a moderate reduction in lipid droplet size and fat lobule area compared to the vehicle controls (Figure 1C). These changes were markedly more pronounced in eWAT, resulting in noticeable necrotic areas. The widespread lack of perilipin protein in these cells (Figure 1D) suggests that they are only lipid droplets, i.e. dead adipocytes present with a concomitant infiltration of immune cells. Therefore, tamoxifen induces a high level of fat loss in sWAT and particularly eWAT due to widespread necrosis of adipocytes.

### 3.2 PPARγ deletion exacerbates tamoxifen-induced fat loss

**Figure 2:**
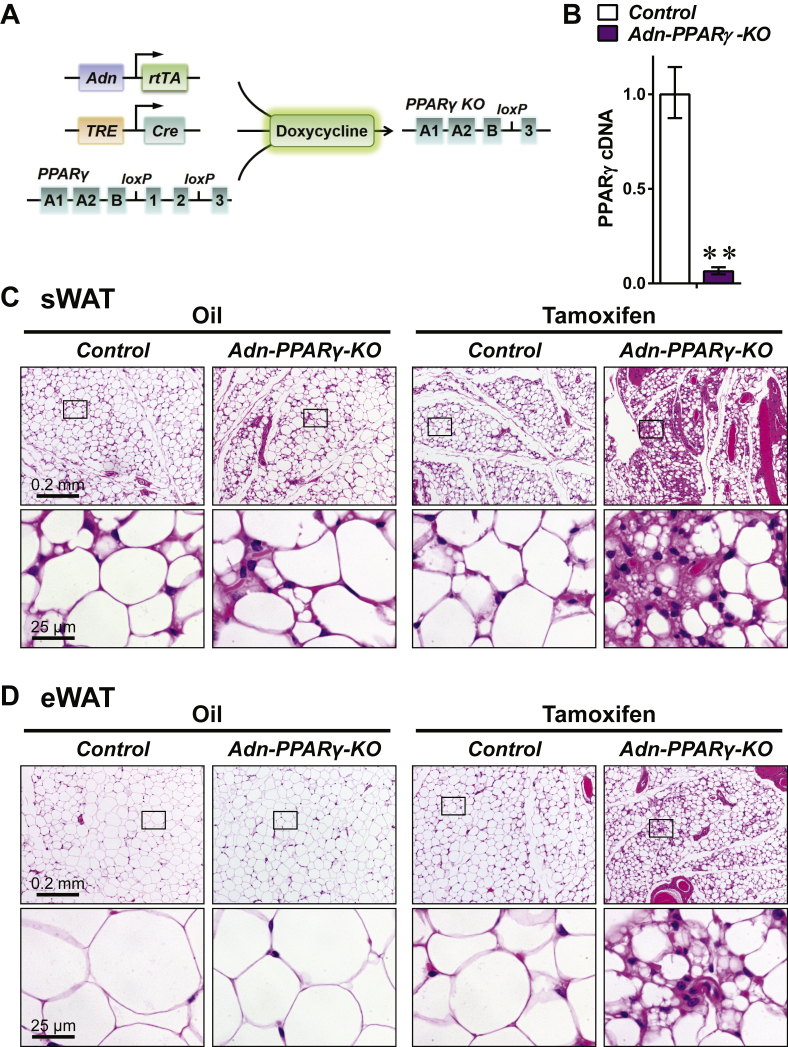
**Adipocyte-specific PPARγ knockout aggravates the fat loss induced by tamoxifen**. *Adn-PPARγ-KO* ([*Adn-rtTA; TRE-Cre; PPARγ*^*flox/flox*^]) and control mice ([*Adn-rtTA; PPARγ*^*flox/flox*^]) were fed doxycycline diet for 5 consecutive days to eliminate PPARγ in adipocytes. **(A)** A schematic drawing of the doxycycline-inducible knockout of the *PPARγ* gene in *Adn-PPARγ-KO* mice. The *Adn-rtTA* transgene expresses the reverse tetracycline transcription activator (rtTA) protein specifically in adipose tissue. It binds to the tetracycline response element (*TRE*) promoter and activates the expression of Cre recombinase, only in the presence of doxycycline. Thus, the Cre-mediated knockout of PPARγ is exclusively in adipocytes, and under temporal control of doxycycline administration. **(B)** RT-qPCR of *PPARγ* mRNA in adipose tissue after doxycycline diet. Data are presented as the mean ± SEM. n = 11 (*Control*) and 20 (*Adn-PPARγ-KO*). **p < 0.01. **(C and D)** Concomitantly with doxycycline diet, mice were subjected to 5 daily oral gavages of tamoxifen (100 mg/kg BDW/day) or vehicle oil at a matched volume (5 mL/kg BDW/day). On day 5, the surviving fat pads of sWAT **(A)** and eWAT **(B)** were dissected and processed for H&E staining. Lower panels show magnified views of the boxed areas in upper panels.

As a master transcription factor for adipogenesis, PPARγ was reported to be required for survival of mature fat cells [13], [20]. Tamoxifen-inducible knockout of PPARγ in adult adipocytes led to acute necrosis in fat pads [21]. To dissect the roles of PPARγ and tamoxifen in adult fat survival, we employed a doxycycline-inducible, adipocyte-specific PPARγ knockout mouse model (*Adn-PPARγ-KO*) [18]. We have crossed three individual mouse strains, *Adn-rtTA* [16], *TRE-Cre* [12], and *PPARγ*^*flox/flox*^ [13]. *Adn-PPARγ-KO* mice allow for PPARγ deletion specifically in adipocytes, only after doxycycline treatment (Figure 2A). Adult mice were subjected to doxycycline chow diet for 5 consecutive days to deplete PPARγ acutely (Figure 2B), accompanied by oral gavages of tamoxifen or vehicle (oil). Consistent with our previous observations (Figure 1B–D), tamoxifen treatment resulted in widespread fat cell death in both *Adn-PPARγ-KO* and control ([*Adn-rtTA; PPARγ*^*flox/flox*^]) mice (data not shown). To discern the effects of PPARγ knockout, we compared the histology of the surviving fat lobules between the two genotypes (Figure 2C and D), and observed that PPARγ depletion synergistically exacerbated the fat loss and adipocyte death effects from tamoxifen. This exacerbation was more prominent in sWAT (Figure 2C) than in eWAT (Figure 2D), consistent with the fat pad preference reported by Evans and colleagues [13]. The adipocyte survival after PPARγ removal was significantly deteriorated when combined with the tamoxifen treatment, suggesting a form of “synthetic lethality” with the combination treatment of tamoxifen and the elimination of a specific key adipogenic transcription factor.

### 3.3 Tamoxifen treatment leads to *de novo* adipogenesis differentially in sWAT and eWAT

**Figure 3:**
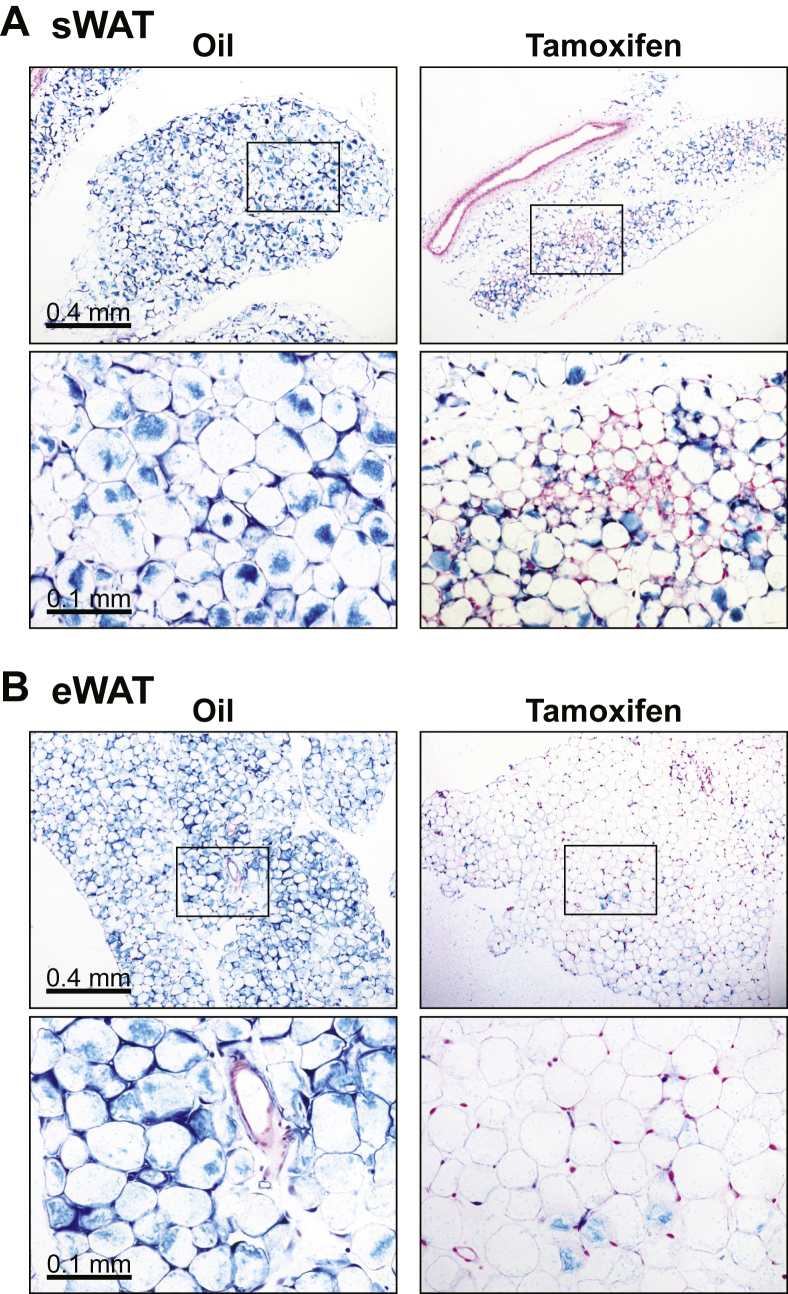
***De novo* adipogenesis in fat pads recovered from tamoxifen treatment**. AdipoChaser mice ([*Adn-rtTA; TRE-Cre; Rosa26-loxP-Stop-loxP-lacZ*]) were first fed on doxycycline diet for 1 week to induce β-galactosidase expression in all mature adipocytes, and then switched to regular diet for 1 week to wash out doxycycline-induced Cre expression. Subsequently, mice were subjected to 5 daily oral gavages of tamoxifen (100 mg/kg BDW/day) or vehicle oil at a matched volume (5 mL/kg BDW/day). 10 days after the last tamoxifen administration, the recovered fat pads of sWAT **(A)** and eWAT **(B)** were stained for β-galactosidase activity (blue) and counterstained with Nuclear Fast Red (red). Lower panels show magnified views of the boxed areas in upper panels.

In light of the fat mass restoration after tamoxifen withdrawal (Figure 1A and B), we monitored the genesis of the replenished adipocytes taking advantage of the AdipoChaser mouse model ([*Adn-rtTA; TRE-Cre; Rosa26-loxP-Stop-loxP-lacZ*]) that we developed previously [10]. After a complete and specific labeling of adipocytes by exposure to doxycycline-containing diet for one week, mice were fed on regular diet for an additional week to thoroughly wash out the Cre recombinase expression and its labeling activity. Mice were then subjected to tamoxifen treatment for 5 days using the standard gavage protocol described above and a subsequent washout for 10 days. After the fat mass recovery, we observed small clusters of β-galactosidase (LacZ) negative adipocytes with multilocular lipid depots in sWAT, indicating that they resulted from *de novo* differentiation during the recovery period and not from pre-existing adipocytes (Figure 3A). All adipocytes in oil-treated controls remained LacZ-positive, excluding the possibility of fat cell turnover unrelated to tamoxifen treatment. Strikingly, most of the adipocytes in tamoxifen-treated eWAT were LacZ-negative (Figure 3B), suggesting a major turnover. The differential *de novo* adipogenesis in sWAT and eWAT was consistent with the tamoxifen-induced fat loss (Figure 1B–D). Our findings highlight the possibility that tamoxifen exposure *per se* induces *de novo* differentiation of adipocytes. If the focus of a given study is on the process of adipogenesis, the tamoxifen-induced adipocyte turnover may lead to artifactual changes that would not be seen under normal physiological conditions and can interfere with studies on adipose pathophysiology and lineage tracing.

### 3.4 Prolonged existence and nuclear translocation activity of tamoxifen in adipose tissue

**Figure 4:**
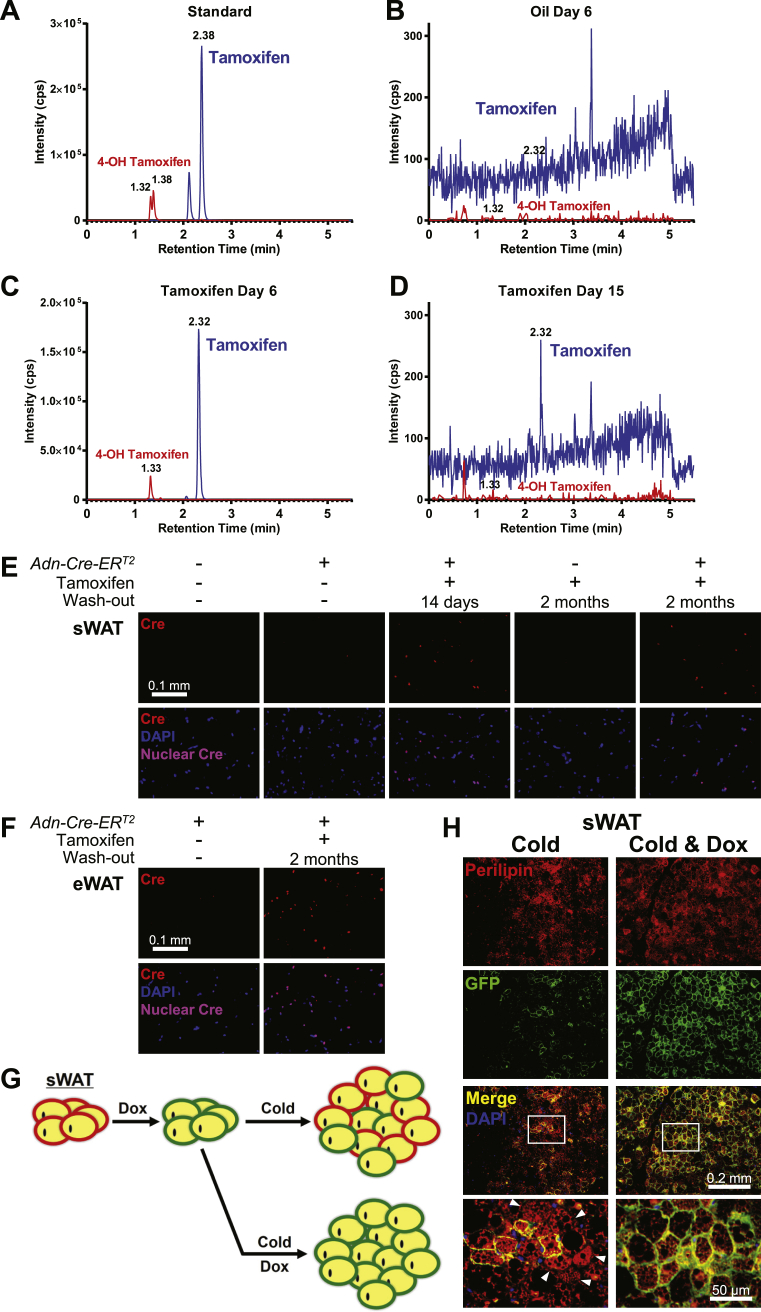
**Residual tamoxifen extends nuclear translocation of Cre-ER**^**T2**^. **(A to D)** Qualitative mass spectrometry of tamoxifen and 4-hydroxytamoxifen (4-OH tamoxifen) in adipose tissue of mice after 5 daily oral gavages of tamoxifen (100 mg/kg BDW/day) or vehicle oil at a matched volume (5 mL/kg BDW/day). The approximate retention times of tamoxifen and 4-OH tamoxifen are indicated by numbers in the plotting area. **(A)** Chemically synthesized standards. **(B and C)** Tissue extract 1 day after the last gavage (day 6) of oil **(B)** or tamoxifen **(C)**. **(D)** Tissue extract 10 days after the last gavage (day 15) of tamoxifen. **(E and F)***Adn-Cre-ER*^*T2*^ mice were subjected to 5 daily intraperitoneal injections of tamoxifen (100 mg/kg BDW/day) followed by indicated washout time. Wildtype male or *Adn-Cre-ER*^*T2*^ female mice without tamoxifen treatment served as negative controls. Paraffin sections of sWAT **(E)** and eWAT **(F)** were subjected to immunofluorescence for Cre recombinase. Upper panels: Cre signal. Non-specific fluorescence level in wildtype control was uniformly subtracted from all the images. Lower panels: Cre signal merged with DAPI staining. **(G and H)** [*Adn-rtTA; TRE-Cre; Rosa26-mT/mG*] mice were fed on doxycycline diet for 10 days to induce GFP expression in all mature adipocytes, and then switched to regular diet for 3 days to wash out doxycycline-induced Cre expression. Subsequently, mice were subjected to 10 days of cold exposure, concomitantly fed on regular or doxycycline diet. **(G)** A schematic model of lineage tracing results supporting *de novo* differentiation of beige adipocytes. The initial doxycycline administration irreversibly switches the expressed reporter in mature adipocytes from tdTomato (red) to GFP (green). After doxycycline washout followed by cold exposure, GFP^-^tdTomato^+^ (red) beige adipocytes are from *de novo* differentiation, while GFP^+^tdTomato^−^ (green) cells are progeny of the pre-existing mature adipocytes. If doxycycline is administrated during cold exposure, all the *de novo* differentiated beige cells are irreversibly switched to GFP expression. **(H)** Paraffin sections of sWAT were subjected to co-immunofluorescence of perilipin (red) and GFP (green). The lowest panels show magnification of the boxed areas in the merged images. Arrowheads: GFP-negative multilocular adipocytes.

Powers and colleagues reported the prolonged Cre-loxP recombination in pancreatic islets of a tamoxifen-inducible mouse model [5]. These were very valuable observations that prompted us to examine the situation more closely in the context of the adipocyte as well. We determined whether this unanticipated phenomenon is related to the partitioning of tamoxifen into the lipid droplets of adipocytes from where it could leak out very slowly over time, in which case the lineage tracing results would be very challenging. We performed a preliminary mass spectrometry analysis to examine the presence of tamoxifen and its active metabolite, 4-hydroxytamoxifen, in adipose tissue (Figure 4A–D). While neither tamoxifen nor 4-hydroxytamoxifen stood out from the background noise in the vehicle-treated controls (Figure 4B), both were detected at a prominent intensity 1 day after the 5-day tamoxifen treatment period (Figure 4C), and remained detectable 10 days later (Figure 4D).

To determine whether the levels of residual tamoxifen retain the ability to convey nuclear translocation activity for the Cre-ER^T2^ fusion protein, we subjected the transgenic mouse strain *Adn-Cre-ER*^*T2*^ [14] to tamoxifen treatment. By immunofluorescence, we can detect the concentrated Cre-ER^T2^ protein in the nucleus. However, if the fusion protein is spread throughout the cytosol, it gives rise to a diffuse label of very low intensity and is difficult to visualize. To our surprise, the nuclear signal in both sWAT (Figure 4E) and eWAT (Figure 4F) was sustained not only for two weeks post washout, but it could also be detected through two months post tamoxifen treatment. Taken together, our findings support that tamoxifen remains present and active in adipose tissue for an extended period, effectively translocating the Cre recombinase into the nucleus. While this may be a desirable effect if the goal is simply to eliminate inducibly a genomic locus flanked by loxP sites, tamoxifen-based systems may be challenging to operate in the context of lineage tracing studies in adipose tissue due to the inability to effectively wash out the ligand.

The prolonged activity of tamoxifen could be an explanation for the inconsistent results from lineage tracing experiments [10], [11]. To circumvent the potential artifacts from tamoxifen, we employed the tetracycline-on transcription system for our lineage tracing studies. We generated the [*Adn-rtTA; TRE-Cre; Rosa26-mT/mG*] mouse model and labeled the adipocytes with green fluorescence protein (GFP) expression after 10 days of doxycycline diet, followed by 3 days of regular diet to wash out doxycycline. So the pre-existing adipocytes are labeled green. As one of the applications, we subjected the mice to cold exposure and traced the origin of the emerging beige adipocytes in sWAT (Figure 4G). Compared to the control mice fed a doxycycline diet throughout the cold exposure, mice fed a regular diet without dox showed a marked increase in the GFP-negative, multilocular cell population, supporting *de novo* adipogenesis as a contributing mechanism to beige cell generation (Figure 4H). The presence of GFP-positive multilocular cells supports mature adipocyte conversion as a concomitant mechanism, occurring at a low frequency. These results were consistent with our previous findings with the LacZ reporter [10], demonstrating that the majority of beige fat cells that emerge upon an initial cold exposure of the mice arise through a *de novo* differentiation pathway.

## 4 Discussion

As one of the most widely used strategies for temporal control of genetic manipulation, tamoxifen, combined with the Cre-ER^T2^/loxP transgenic system, has been heavily employed for lineage tracing studies [1]. In this context, the experimental doses of tamoxifen for mice are hundreds of times higher than those employed for human breast cancer therapy. For the first time, we characterize the profound effects of this supraphysiological tamoxifen exposure on adipose physiology and lineage tracing experiments. Tamoxifen treatment at a typical oral dosage (5 days of 100 mg/kg BDW/day) results in acute lipoatrophy followed by *de novo* adipogenesis and recovery in fat mass. The fat cell turnover is much more dramatic in eWAT relative to sWAT, and has the potential to be further synergistically aggravated in the context of additional genetic manipulations. As an example, we demonstrate the interaction of tamoxifen with the elimination of PPARγ in adipocytes. Surprisingly, residual tamoxifen remains detectable in adipose tissue 10 days post tamoxifen treatment, and its nuclear translocation activity for the Cre-ER^T2^ fusion protein lasts beyond two months. This long systemic presence is not exclusive to adipose tissue but was previously reported for pancreatic β-cells as well [5]. In the case of the β-cells, this could also be a phenomenon of slow release from lipid droplets in adipose tissue. Hence our findings call for caution to tamoxifen usage in adipose studies, especially lineage tracing.

Our data indicate that an extended period is necessary to completely wash out the tamoxifen activity in adipose tissue. For instance, to trace the origin of cold-induced beige adipocytes in sWAT, a 10-day washout time may not be long enough to exclude the false positive cells in reporter expression [11]. Considering that the 1.5‰ residual tamoxifen detected in adipose tissue 10 days post treatment (Figure 4C and 4D) confers nuclear translocation of Cre-ER^T2^ protein beyond two months (Figure 4E and F), reducing the dose of tamoxifen treatment may not be an effective strategy to prevent its prolonged activity. Moreover, in lineage tracing experiments requiring high-percentage labeling of specific cell populations, a supraphysiological dose of tamoxifen is necessary for the complete labeling efficiency. In contrast, our experiments with the accurate temporal control offered by doxycycline, which can be completely washed out overnight, unmask the reporter-negative cells and *de novo* differentiation as a contributing mechanism [10]. Here we confirm this finding with a fluorescence reporter (Figure 4H), excluding the possibility of an artifactual washout of the β-galactosidase stain. This point is further strengthened by the positive controls with concomitant doxycycline-induced recombination throughout the experiment that gives rise to uniform labeling. Unquestionably, doxycycline has side effects as well due to low level mitochondrial toxicity and its impact on the gastrointestinal flora due to the fact that it is an antibiotic [22]. There is no perfect system currently available for inducible manipulation of gene expression *in vivo*. Nevertheless, in light of its rapid clearance, effective recombination, and the fact that it does not cause any cell death in adipocyte [10], we believe it is a superior system for lineage tracing studies in the adipocyte.

Importantly, for other applications that do not focus on lineage tracing experiments, but rather use tamoxifen-induced Cre activity to eliminate gene products in the mature adipocyte, the use of this system may very well be legitimate, as long as it is controlled with all experimental groups receiving tamoxifen.

Interestingly, the supraphysiological tamoxifen (∼100 mg/kg BDW/day) depletes the fat depot preferentially in eWAT, similar to the body fat distribution directed by estrogen [7] and ERα [8]. Whether tamoxifen confers this acute lipoatrophy through ERs awaits further investigation, e.g. utilizing the ERα or ERβ knockout mice [9]. A recent report suggested the production of reactive oxygen species as part of the mechanism [23]. The lipoatrophic effect of tamoxifen does not depend on the approach of oral gavage, since intraperitoneal injections at comparable doses also induces fat loss [23]. In seemingly contrast, clinical tamoxifen treatment (∼0.5 mg/kg BDW/day) for breast cancer patients exhibited a long-term adipogenic effect [24], especially in visceral fat [25]. The increased adiposity associated with metabolic complications such as liver steatosis [25] and augmented serum leptin levels [26], [27]. Based on our findings, it is tempting to speculate that the long-term adipogenesis may be a compensatory response to tamoxifen-induced lipoatrophy and draws attention to possible metabolic side effects from tamoxifen exposure in cancer patients.

## 5 Conclusions

In summary, we demonstrate a pro-death effect on adipocytes, and a surprisingly long presence and nuclear translocation activity of tamoxifen in adipose tissue. This calls for caution when tamoxifen is employed for temporal control of gene rearrangements and lineage tracing studies.

## Author contributions

R.K.G. and P.E.S. conceptualized the study. R.Y., Q.A.W., J.G.M., R.K.G. and P.E.S. designed the experiments and analyzed the data. R.Y., Q.A.W., C.T., M.S. and L.V. performed the experiments. R.Y. and P.E.S. wrote the manuscript.
